# miRNAs in Sera of Tunisian patients discriminate between inflammatory breast cancer and non-inflammatory breast cancer

**DOI:** 10.1186/2193-1801-3-636

**Published:** 2014-10-28

**Authors:** Khouloud Hamdi, David Goerlitz, Neila Stambouli, Mohammed Islam, Olfa Baroudi, Bilel Neili, Farhat Benayed, Simon Chivi, Christopher Loffredo, Irene A Jillson, Amel Benammar Elgaaied, Jan K Blancato, Raja Marrakchi

**Affiliations:** Laboratory of Genetics, Immunology and Human Pathology, Faculty of Sciences, University of Tunis El Manar, El Mannar I, Tunis, 2092 Tunisia; Lombardi Comprehensive Cancer Center, Georgetown University Medical Center, Washington, DC 20007 USA; Department of Medical Oncology, Hannibal International Clinic, Les Berges du Lac 2, Tunis, Tunisia

**Keywords:** Breast cancer, Inflammatory breast cancer, microRNA, Circulating microRNA, Prognostic biomarkers, RT-qPCR

## Abstract

In recent years, circulating miRNAs have attracted interest as stable, non-invasive biomarkers for various pathological conditions. Here, we investigated their potential to serve as minimally invasive, early detection markers for inflammatory breast cancer (IBC) and non-inflammatory breast cancer (non-IBC) in serum. miRNA profiling was performed on serum from 20 patients with non-IBC, 20 with IBC, and 20 normal control subjects. Real-time reverse transcription-polymerase chain reaction (qRT-PCR) was applied to measure the level of 12 candidate miRNAs previously identified in other research(miR-342-5p, miR-342--3p, miR-320, miR-30b, miR-29a, miR-24, miR-15a, miR-548d-5p, miR-486-3p, miR-451, miR-337-5p, miR-335).We found that 4 miRNAs (miR-24, miR-342-3p, miR-337-5p and miR-451) were differentially expressed in serum of IBC patients compared to non-IBC, and 3 miRNAs (miR-337-5p ,miR-451and miR-30b) were differentially expressed in IBC and non-IBC patients combined compared to healthy controls. miR-24, miR-342-3p, miR-337-5p and miR-451 were found to be significantly down-regulated in IBC patients compared to non-IBC. Likewise, the expression level of mir-451 showed significant down-regulation in IBC serum, while mir-30b and miR-337-5p were up-regulated in non-IBC serum comparatively to normal controls. Using receiver operational curve (ROC) analysis, we show that dysregulated miRNAs can discriminate patients with IBC and non-IBC from healthy controls with sensitivity ranging from 76 to 81% and specificity from 66 to 80%, for three separate miRNAs. In conclusion, our data suggest that circulating miRNAs are potential biomarkers for classifying IBC and non-IBC, and may also be candidates for early detection of breast cancer.

## Background

Breast cancer (BC) is the most commonly diagnosed cancer in women worldwide. In Tunisia, this tumor represents the leading cause of cancer death among women, accounting for 30% of all malignancies in women and with a particularly high proportion of inflammatory types of breast cancer (Belkacémi et al. 2010).

Inflammatory breast cancer (IBC) is rare but the most lethal form of locally advanced breast carcinoma, occurring in 1% to 6% of all invasive breast tumors. (Lehman et al. 2012); affected patients have a poor prognosis due to infiltration of tumor emboli that metastasize within the dermal lymphatic vessels of the skin overlying the breast (Boussen et al. 2010). According to the American Joint Committee on Cancer (AJCC), IBC is “a clinico-pathologic entity characterized by diffuse erythema and edema (i.e., peau d’orange) of the breast, often without an underlying palpable mass” (Lehman et al. 2012). Furthermore, the AJCC’s staging scheme defines IBC as T4d tumor at diagnosis, and characterizes it as typically affecting young women, often during their childbearing years (Laere et al. 2005). At diagnosis, a majority of patients have axillary lymph nodes involved, and one third present with distant metastases (Bertucci et al. 2005).

Gene expression profiles provide specific molecular signatures containing information which may enable explanation of the mechanisms of tumor development and progression and the poor prognosis observed in IBC. Indeed, several studies comparing IBC to non-IBC have demonstrated a specific mRNA expression signature in IBC that is significantly enriched for genes involved in cell motility, inflammation, immune response and stem cell biology (Bertucci et al. 2004; Ben Hamida et al. 2008). Furthermore, expression profiling performed by Marrahchi et *al.* have further strengthened the evidence of the role of WISP3 as a tumor suppressor gene and RhoC as an oncogene which act in concert to give rise to the highly aggressive IBC phenotype in the Tunisian population. (Marrakchi et al. 2009).

Some studies have noted the dysregulation of several miRNA in IBC tumors, but (Iorio et al. 2005; Blenkiron et al. 2007; Auwera et al. 2010), no study has focused on miRNA levels in sera of patients with IBC. The aim of this study was to identify a molecular signature of IBC based on miRNA profile in serum and to use this profile as an early diagnosis criterion. To this end, we present the first investigation of a panel of miRNA in the serum of inflammatory breast cancer patients and compared this profile to non-inflammatory breast cancer cases and non-cancer controls.

## Materials and methods

### Ethics statement

Written informed consent was obtained from all participants involved in this study. This study was approved by the Georgetown University Institutional Review Board and the Ethical Committee of Pasteur Institute of Tunis, Tunisia.

### Serum samples and clinical data

In this study, a total of 20 women with non-IBC, 20 women with IBC, and 20 cancer-free women were recruited. Peripheral blood samples (5 ml per subject) were obtained from each study subject and were collected between March 2011 and January 2012 from patients being diagnosed and treated at Hannibal International Clinic of Tunis at the time of diagnosis before they underwent any therapeutic procedure. Control blood samples were collected at the same period from healthy women volunteers with no history of malignant diseases, no blood donations received in the previous 3 years, and no current inflammatory conditions; they were matched to the cases on menopause status. The blood tube was centrifuged at 1200 × g for 10 min at 4°C followed by careful separation of serum. Another centrifugation was performed for 10 min at 10,000 × g at 4°C to remove the cellular debits. The serum was stored at −80°C until it was used for RNA extraction.

The clinico-pathological data and tumor characteristics of the patients at time of diagnosis are illustrated in Table 1. We classified the cancer stage according to the TNM staging system of the seventh edition of the AJCC Cancer Staging Manual. There were no statistical differences in age, estrogen receptor, progesterone receptor, and Her2/*neu* between the two patients groups. Patient histopathology results were confirmed by the pathologist’s examination of the surgical resection.

**Table 1:** **Clinicopathological features of inflammatory breast cancer (IBC), non-IBC breast cancer patients and controls**

Patient characteristics	IBC		Non-IBC		Healthy	
	N = 20	(% )	N = 20	(%)	N = 20	(%)
Age range	34-70		29-78		21-80	
Mean age	49.0		50.2		45.5	
**Menopausal status**						
Pre-menopausal	10	50%	10	50%	10	50%
Post-menopausal	10	50%	10	50%	10	50%
**Histological grade**						
I	0	0%	0	0%		
II	9	45%	7	35%		
III	11	55%	13	65%		
**Node status**						
N0	2	10%	7	35%		
N1	16	0%	13	65%		
N2	2	10%	0	0%		
N3	0	80%	0	0%		
**Tumor**						
T1	0	0%	0	0%		
T2	0	0%	6	30%		
T3	0	0%	13	65%		
T4	20	100%	1	5%		
**Metastasis**						
M0	14	70%	4	20%		
M1	0	0%	2	10%		
Unknown	6	30%	14	70%		
**ER status**						
Positive	3	15%	11	55%		
Negative	17	85%	9	45%		
**PR status**						
Positive	4	20%	11	55%		
Negative	16	80%	9	45%		
**HER2 status**						
Positive	13	65%	13	65%		
Negative	7	35%	7	35%		

### RNA extraction and reverse transcription

Total RNA was extracted from 500 μl serum using mirVana ™PARIS™Kit from Life Technologies according to manufacturer’s protocol. Briefly, 500 μl of 2× Denaturing Solution was added to each sample. Total RNA was extracted with acidic phenol:chloroform. The aqueous phase was loaded onto the Filter Cartridge after the addition of 1.25 volume of 100% ethanol. After several washing processes, the RNA was eluted into 60 μl of RNase-free water. The RNA concentration and purity were controlled by UV spectrophotometry using a Nanodrop ND-1000 a verifier (Thermo Scientific). The RNA specimens were stored at − 80°C until reverse transcription.The corresponding cDNAs were made using TaqMan MicroRNA reverse transcription (RT) reagents and specific primers for the miRNAs (Applied Biosystems). The amount of RNAs used for RT was 2 ng and the cDNA specimens were stored at − 20°C until PCR.

### miRNA quantification by real-time quantitative PCR

Real-time PCR was performed for each of 13 miRNAs identified in previous research, as described above (hsa-miR-5p, hsa-miR-3p, hsa-miR-320, hsa-miR-30b, hsa-miR-29a, hsa-miR-24, hsa-miR-15a, hsa-miR-548d-5p, hsa-miR-520a, hsa-miR-486-3p, hsa-miR-451, hsa-miR-337-5p, hsa-miR-335). For the 20 μl reaction, 1.33 μl cDNA was combined with 10 μl of TaqMan® Universal PCR Master Mix II (2×), without uracil-N-glycosylase, 1 μl of the TaqMan miRNA assay, and 7.67 μl of nuclease-free water. The reaction mixtures were incubated at 95°C for 10 min, followed by 40 cycles of 95°C for 15 s and 60°C for 1 min.All assays were performed on the Applied Biosystems 7900 HT Sequence Detection System following the manufacturer’s protocol. For each sample, the miRNA expression levels were assayed in triplicate and were run with endogenous control RNU-48 (small nuclear RNA) also in triplicate, with a single no template negative control reaction (seven reactions total per sample per miRNA), and were calculated using the 2^− ∆∆Ct^method (Livak & Schmittgen 2001). The cycle threshold (Ct) values were calculated as the number of cycles needed for the fluorescence to reach a specific threshold level of detection.

### Statistical methods

The miRNA data were analyzed using the programming language R based on the methodology described by Livak et al. For each miRNA, we calculated the average cycle threshold difference (∆Ct) across multiple samples within a groupthe fold change between two groups, and the statistical significance of the observed difference between two groups (p-value). Statistical significance of the change in expression between the two groups (BC vs. IBC) is determined by using the two-sample t-test assuming unequal variance.

Receiver operating characteristic (ROC) curves and the area under the ROC curve (AUC) were used to evaluate the diagnostic effects of the miRNAs and to determine appropriate cut-off points.

The sensitivity and specificity of the optimum cut-off point were defined as those values that maximized the area under the ROC curve. All statistical analyses were performed using SPSS 15.0 software, and p value < 0.05 was considered statistically significant.

## Results

### miRNA expression profile of IBC, non-IBC, and healthy controls

Among the 12 miRNAs analyzed, 5 miRNAs were differentially expressed in the serum of the 3 groups of subjects (Table 2), using the criteria of at least two-fold differential expression and significance level of < 0.05. Regarding the comparison of IBC patient samples to healthy controls we found that only miR-451 was significantly dysregulated (*p* = 0.019), showing significant down-regulation (15.8-fold) in IBC sera comparison to healthy controls. In comparing non-IBC sera with normal sera we observed that mir-337-5p and mir-30bwereover expressed in non-IBC (*p* = 0.049; 16.8-fold increase, and *p* = 0.032; 14.4 folds increase, respectively). In terms of expression levels of miRNAs in IBC sera compared to non-IBC sera, we found that miR-24, miR-342-3p, miR-337-5p and miR-451 were differentially expressed among these, mir-451 was significantly down-regulated (57.5-fold reduction) in IBC serum compared non-IBC serum (*p* = 0.000), while, miR-337-5p was up-regulated in non-IBC serum compared to IBC serum (p = 0.025, 12.3-fold difference). Likewise, mir-342-3p and mir-24 levels were higher in non-IBC (10.5-fold; *p = 0.047,* and 16-fold, *p* = 0.016, respectively.

**Table 2:** **Differentially expressed miRNAs obtained from comparison of subjects with IBC or non-IBC compared to healthy controls**

miRNAs	IBC serum Mean ± SD log2 (fold change value)	Non-IBC serum Mean ± SD log2 (fold change value)	***P*** value	Healthy serum Mean ± SD log2 (fold change value)	IBC serum Mean ± SD log2 (fold change value)	***P*** value	Healthy serum Mean ± SD log2 (fold change value)	Non-IBC serum Mean ± SD log2 (fold change value)	***P*** value
has-mir-24	-7.4	-3.4	0.016	-6.1	-7.4	NS	-6.1	-3.4	NS
has-mir-342-3p	-5.9	-2.6	0.047	-5.5	-5.9	NS	-5.5	-2.6	NS
has-mir-451	-11.8	-5.9	0.000	-7.8	-11.8	0.019	-7.8	-5.9	NS
has-mir-30b	-4.8	-2.7	NS	-6.7	-4.8	NS	-6.7	-2.7	0.049
has-mir-337-5p	-3.1	0.5	0.025	-3.4	-3.1	NS	-3.4	0.5	0.032
has-mir335	-4.9	-2.5	NS	-4.9	-4.9	NS	-4.9	-2.5	NS
has-mir-320	−9.6	-6.3	NS	-7.2	-9.6	NS	-7.2	-6.3	NS
has-mir-548-5d	-0.6	0.0	NS	-2.3	-0.6	NS	-2.3	0.0	NS
has-mir486-3p	-3.4	-1.7	NS	-4.0	-3.4	NS	-4.0	-1.7	NS
has-mir-342-5p	-2.6	-0.8	NS	-4.0	-2.6	NS	-4.0	-0.8	NS
has-mir-15a	-4.2	-2.6	NS	-4.3	-4.2	NS	-4.3	-2.6	NS
has-mir-29a	-6.6	-4.6	NS	-5.2	-6.6	NS	-5.2	-4.6	NS

### Profiles of individuals based on the expression level of 12 miRNAs studied

**Figure 1:**
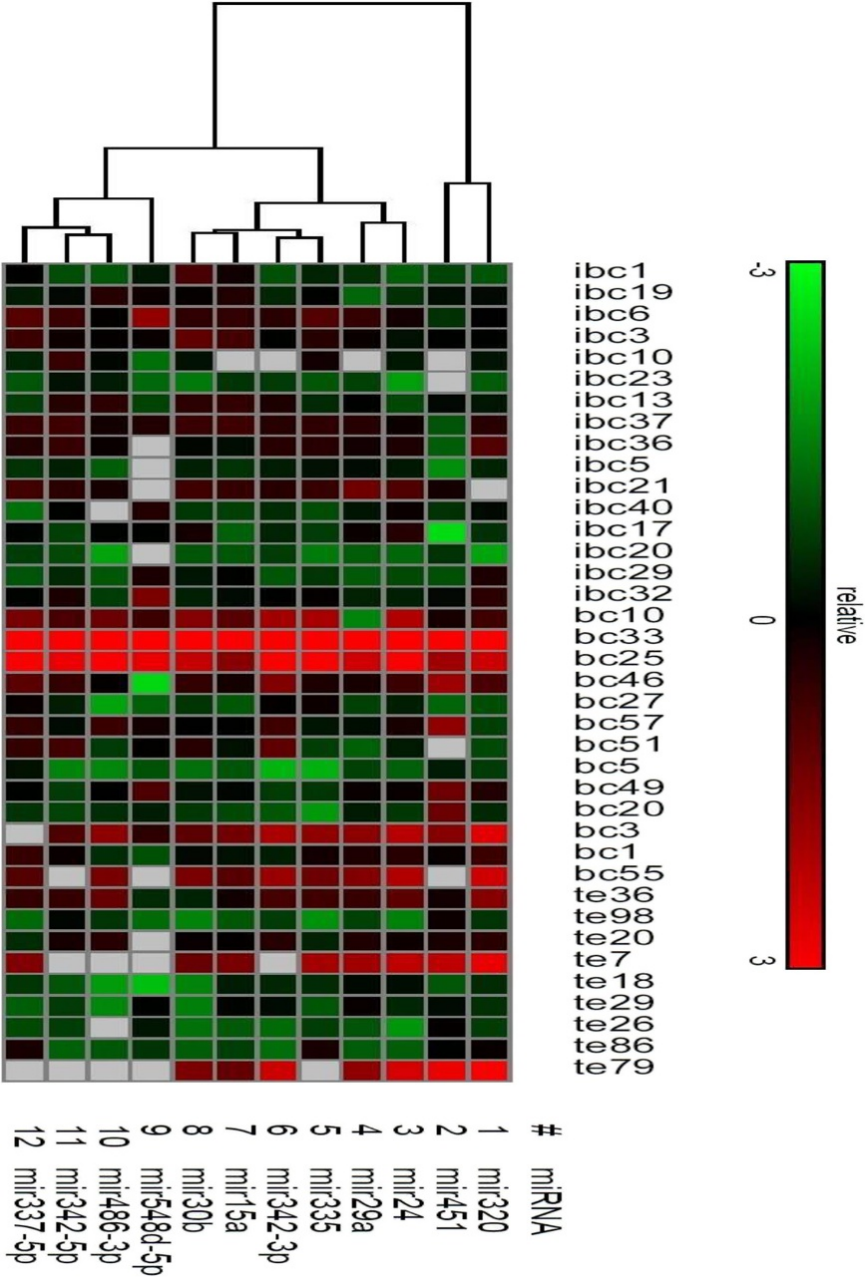
**Hierarchical clustering of miRNAs expression.** miRNAs profiles of 17 IBC, 12 non-IBC and 9 healthy controls were clustered based on the 3 groups (IBC, non-IBC and healthy controls). Samples are in columns, miRNAs in rows. High expression values are indicated by bright red shades, low expression values are shown in green.

The clustering of miRNA expression profiles from the three groups of subjects is shown in Figure 1. In each group; the levels of the miRNAs are positively correlated. In contrast the results of the heat map that was constructed for the 12 measured miRNAs demonstrated a diversity of expression profiles, with 9 different profiles for healthy controls, 17 for IBC patient sera, and 12 for non-IBC. A downward trend of miRNAs is observed in the IBC subjects; while in non-IBC sera a tendency to over expression was observed. However, no clear clusters corresponding to the three groups were evident. In the control group, where most of the profiles were associated with low miRNA expression, two profiles with high expression were also observed and are shown in Figure 1.

### Evaluation of mir-30b, mir-337-5p and mir-451 in plasma as potential prognosis markers

**Figure 2:**
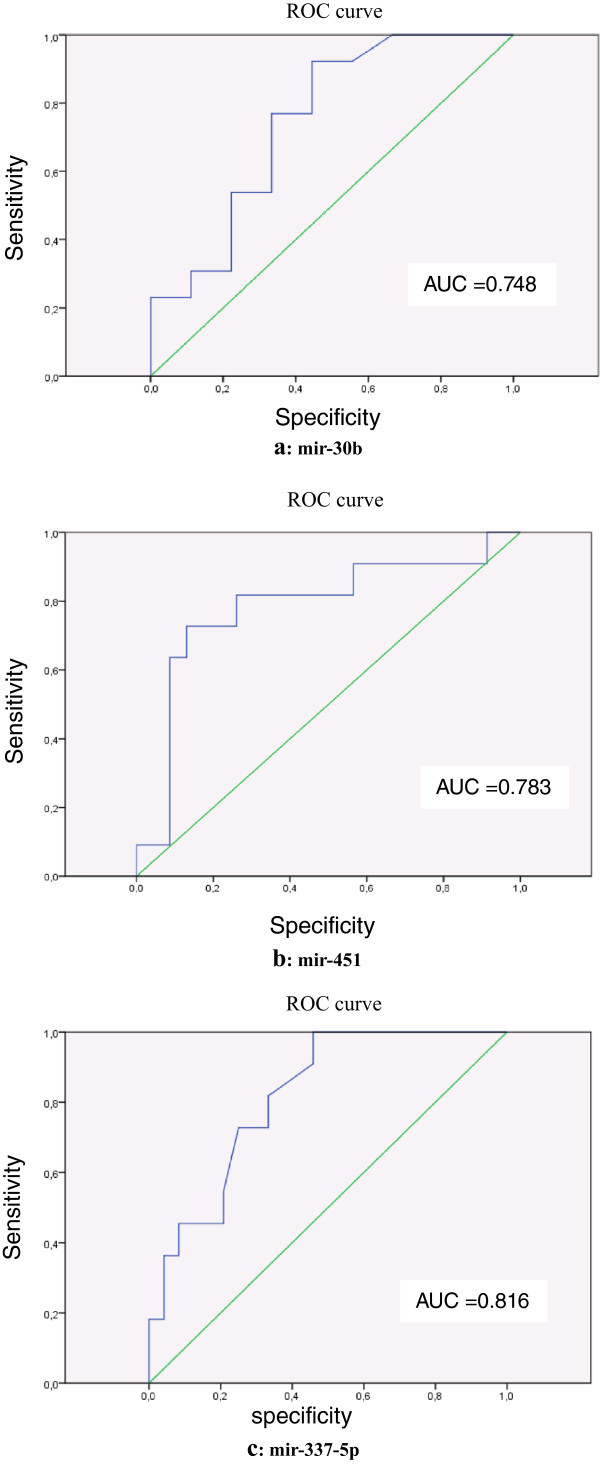
**Evaluation of mir-30b, mir-451 and mir-337-5p expression in IBC and non-IBC serum by receiver operating characteristics (ROC) curve analysis.** **Fig** 2 **(a)**: Evaluation of mir-30b expression in non-IBC serum by curve ROC. **Fig** 2 **(b)**: Evaluation of mir-451 expression in IBC serum by curve ROC. **Fig** 2 **(c)**: Evaluation of mir-337-5p expression in non-IBC serum by ROC curve.

To evaluate whether these serum miRNAs can be used as potential diagnostic markers for IBC or non-IBC, receiver-operating characteristic (ROC) curve analyses were performed.mir-30b yielded an AUC of 0.748 with 76% sensitivity and 66% specificity in discriminating non-IBC from a cut-off value of −16.8 (Figure 2a), while mir-337-5p yielded AUC of 0.816 with 81% sensitivity and 66% specificity in differentiating non-IBC from normal controls, from a cut-off value of −14.4 (Figure 2c). Likewise, mir-451 yielded an AUC of 0.783 with 81% sensitivity and 80.0% specificity in discriminating the IBC subtype from a cut-off value of −57.5 (Figure 2b).

## Discussion

Tumor tissue miRNAs have been demonstrated to be associated with the pathogenesis of breast cancer and proposed to be used as biomarkers for diagnosis and prognosis (Lowery et al. 2008); (Iorio et al. 2005); (Blenkiron et al. 2007). (Giglio et al. 2013). At least 24 candidate oncogenes with gains at the DNA level, discovered with comparative genomic hybridization (CGH) studies, have been reported for IBC in contrast to non-IBC breast tumors. Gene expression studies also support genetic heterogeneity for IBC (Bekhouche et al. 2011).

Although notable progress has been made in the last decade in the study of IBC, early detection is one of the major challenges (Lehman et al. 2012). Within this context, microRNAs (miRNAs), which are non-coding, single-stranded small RNAs, offer a promising new avenue of research since they are responsible for regulating gene expression on the post-transcriptional level. They accomplish this regulation through binding, due to partial or total sequence homology to target mRNAs, thus inhibiting the translation of protein from these mRNAs or by promoting their degradation (Wu et al. 2009). Through this process, miRNAs are involved in the regulation differentiation, metabolism, proliferation and apoptosis (Chen et al. 2012). It has been observed that dysregulated miRNA expression resulted in a wide array of pathological events, including cancer, indicating that miRNAs may become valuable molecular markers for detection and prognosis of tumors. Compared to protein-based biomarkers, miRNA offers several advantages: the complexity of miRNA is much lower than that of proteins; the sequences of many miRNAs are conserved across species; the expression of some miRNAs are restricted to specific tissues or biological stages ; and the levels of miRNAs can also be easily measured by various commonly used laboratory methods (Friedman et al. 2012).

More recently, Mitchell et al., reported that miRNAs existed in serum/plasma, and were originated from cancer tissue (Mitchell et al. 2008). The extreme stability of circulating miRNAs appeared due to their resistance to RNase digestion and to their protection from degradation through inclusion in various protein complexes or membranous particles such as exosomes or microvesicles. The miRNAs are particularly resistant to harsh conditions such as extended storage and multiple freeze-thaw cycles which holds great promise for the use of miRNAs as distinctive, non-invasive cancer biomarkers (Ng et al. 2013);(Blondal et al. 2012).

Since the discovery of circulating miRNAs, numerous studies on miRNA and cancer have been published (Volinia et al. 2006). Focusing on breast cancer, a pilot study performed by Roth C et al. provided the first evidence that tumor-associated circulating microRNAs are elevated in the blood of breast cancer patients and associated with tumor progression (Roth et al. 2010).

Moreover, Heneghan et al. found that expression of miR-195 was significantly elevated in breast cancer patients compared to healthy controls (Heneghan et al. 2010).

The role of serum miRNA in breast cancer has not been thoroughly studied. Our work is based on the hypothesis that circulating miRNAs in serum can potentially serve as novel, minimally invasive biomarkers for early detection of inflammatory and non-inflammatory breast cancer. In the present study, we investigated the potential of 12 circulating miRNAs, selected from previous studies, by analyzing serum profiles of IBC, non-IBC and matched healthy controls. The clustering of miRNA expression profiles that we observed identified four groups of miRNAs that sufficiently discriminated the three groups of patient sera in a blinded fashion.

Moreover, normal sera profiles are rather related to low levels of miRNAs which is in agreement with the fact that most of the miRNAs studied could be considered oncogenes (Du et al. 2013) (Li et al. 2010). However two profiles with high miRNA expression and close to some non-IBC patient sera, are observed in controls leading to a doubt on their normality. In other words such profiles could be associated to the first steps of the disease. Since no clear clusters corresponding to the three serum groups have been carried out, this hypothesis needs more investigation to be confirmed.

Moreover, our results have shown that taken individually some serological mRNA could be pertinent markers of breast cancer. Indeed, we observed that, from the 12 miRNAs studied, five miRNAs (mir-24, mir-342-3p, mir-30b, mir-337-5p and mir-451) were significantly dysregulated in the serum of the 3 groups studied, among which four discriminated between IBC and non-IBC patient sera.

Although serum samples are easily acquired in a relatively noninvasive manner, and isolated miRNAs are readily detected by qRT-PCR, a widely used clinical and laboratory technique, it should be mentioned that miRNA profiles in serum are not necessarily representative of their expression in the tumor. Hence, our results revealing only four discriminative miRNAs between IBC and non-IBC sera are discordant with a previous study on breast tumors that observed 3 times as many discriminative miRNAs. Indeed, since miRNA presence is often observed in healthy subject sera, it is obvious that it may be a consequence of normal physiology. In breast cancer patient serum, this serological expression may be viewed as a cause or a consequence of the oncogenic process. Therefore, if it can be shown prospectively that the levels are over- or under-expressed prior to cancer diagnosis, the determination of the serum miRNA profiles in breast cancer patients may be quite meaningful. In our study, mir-342-3p and mir-24were significantly up-regulated in non-IBC as compared with IBC. This result is in agreement with a previous report on IBC and non-IBC miRNAs expression in tumors, while our results differed in showing a significant up-regulation of miR-337-5p in IBC (Iorio et al. 2005). This up-regulation phenomenon may also be the case for miR-30; moreover the miR-30 family is able to suppress apoptosis (Li et al. 2011) and is up-regulated in non-IBC tumors in comparison with IBC (Iorio et al. 2005)/(Blenkiron et al. 2007). Finally, we also observed that mir-451 was significantly down-regulated in IBC serum as compared with non-IBC and with healthy controls, with adequate specificity and sensibility to serve as a marker for IBC. The lower expression level of mir-451 observed in serum of IBC comparatively to normal sera could be either the consequence of the tumor development or the cause of the disease through more general mechanisms leading to the oncogenic process in IBC. In the last case, mir-451 low expression could be used as an early diagnosis marker of IBC. Our results are in agreement with previous studies showing mir-451 as a tumor suppressor (Li et al. 2013).

In conclusion, if validated in future, larger studies, mir-451 could be considered as a serological marker for IBC, and mir-337-5p and mir-30 for non-IBC, with clear utility in early diagnosis and/or disease prognosis studies. Therefore the continued study of these miRNA dysregulation pathways may lead to breast cancer prevention strategies.

## Authors’ information

Jan Blancato and R Marrakchi are senior authors.
